# Ultrasound is a suitable radiation-free alternative for hip surveillance in children with cerebral palsy or developmental dysplasia of the hip older than one year

**DOI:** 10.1007/s00264-025-06701-2

**Published:** 2025-12-04

**Authors:** Quirin J. Wuermeling, Christian M. Ziegler, Stefanie König, Lara Göttling, Dominic Simon, Sophia S. Goller, Anna-Maria Zvereva, Sebastian A. Schroeder, Alexandra Sitzberger, Nina C. Berger, Thomas R. Niethammer, Boris M. Holzapfel, Ferdinand Wagner

**Affiliations:** 1 Musculoskeletal University Center Munich (MUM), LMU University Hospital, Ludwig-Maximilians-Universität München, Munich, Germany; 2 Department of Radiology, University Hospital, LMU, Munich, Germany; 3 iSPZ Hauner MUC, LMU Munich, Munich, Germany; 4 Clinic for Child Neurology and Social Pediatrics, Child Center Maulbronn, Maulbronn, Germany; 5 Paediatric Orthopaedic Department Klinikum Dritter Orden, Paediatric Orthopaedic Department Klinikum Dritter Orden Munich, Munich, Germany

**Keywords:** Hip ultrasound, Developmental dysplasia of the hip, Cerebral palsy, Hip dislocation

## Abstract

**Purpose:**

Children with chronic diseases are at a significant risk of radiation exposure. This cohort study evaluates the effectiveness and reliability of ultrasonography (US) for detecting femoral head decentration in children with cerebral palsy (CP) and developmental dysplasia of the hip (DDH), comparing it with traditional radiographic techniques to reduce radiation exposure.

**Methods:**

A total of 169 patients were enrolled in the study. Both hips were evaluated in 158 patients, resulting in a total of 327 hips. Patients underwent clinical and radiological assessments, including standardized US. Parameters measured included the ventral and lateral bony and cartilaginous ultrasonographic migration indices (bUMI and cUMI), which were compared with standardized radiographic indices (Reimers index (RI) and extrusion index (EI)).

**Results:**

The lateral bUMI (17.4%) was significantly lower than the lateral cUMI (25.9%). RI values were lower than EI values (16.8% vs. 27.7%). No significant differences were observed between the bUMI and RI, or between the cUMI and EI, indicating the reliability of US. All lateral parameters correlated well with the lateral centre-edge angle (LCE). Positive correlations were found between the lateral cUMI and the radiological indices, with high inter- and intra-rater reliability (ICC). Significant differences in lateral and ventral UMIs were noted when comparing DDH and CP patients.

**Conclusion:**

US is a reliable alternative to radiography for hip surveillance in detecting hip decentration in children with CP and DDH. It reduces radiation exposure while maintaining diagnostic accuracy. The findings support the adoption of US in clinical practice to improve early diagnosis and intervention.

**Supplementary Information:**

The online version contains supplementary material available at 10.1007/s00264-025-06701-2.

## Introduction

Hip surveillance for children with cerebral palsy (CP) integrates clinical and radiographic evaluations guided by a risk assessment associated with gross motor function. This approach aims to counteract the expected progression towards painful hip decentration and functional limitations [1–5]. The balance between potential advantages and disadvantages associated with HS remains intricate and not completely comprehended [6, 7]. Usable radiological imaging, in this case a non-dynamic modality, is dependent on correct positioning techniques, which cannot always be optimally guaranteed in children, especially in severely affected patients. Therefore, imaging often needs to be repeated to ensure it can be analyzed correctly, leading to an even higher exposure to radiation [8]. Additionally, current high-quality publication highlight the fact that children are more prone to radiation side effects than adults [9–12]. Nonetheless, substantial evidence suggests that the benefits of HS for children with CP as a collective may outweigh the harms [13].

The commonly used migration percentage (MP) as proposed by Reimers measures the ratio of the femoral head uncovered by the acetabulum in relation to the head diameter on plain radiographs [14].

In consequence, children are very likely to undergo multiple radiographies in the first two decades of life, in which they are most vulnerable to ionizing radiation. However, substantial evidence from several recent large-scale studies suggests that even low to moderate doses of external ionizing radiation in childhood due to diagnostic imaging increase the risk of malignancy [12, 15–17]. In order to minimize the harm of HS while maximizing its preventive effects, we hypothesized that ultrasound (US) would be suitable to detect the lateral decentration of the femoral head in patients screened or treated for developmental dysplasia of the hip (DDH) or CP. Terjesen and Kay both described methods to depict hip pathologies in patients older than one year – in contrast to the established newborn screening method developed by Graf [18–20]. Terjesen’s method takes metric measurements accurate to the millimeter, while Kay’s method takes a relative measurement, which is preceded by a technically complex software-based measurement [18, 19]. Knowledge of the femoral head diameter is not important for Terjesen’s method. None of the former compared both entities of hip decentration (DDH and CP). Therefore, we utilized and modified the sonographic method described by Hien in order to depict the centration of the femoral head in our patient cohort monitored or treated for CP and DDH [21]. We validated the sonographic migration index (UMI) and compared it with the established radiographic MP [14, 22].

## Methods

### Patient selection

Between October 2020 and December 2021 we enrolled all patients into the study, who visited the outpatient clinic of the paediatric orthopaedic department at Ludwig Maximilians University of Munich and underwent assessment of their hip joints with a.p. pelvic radiographies (supine position) due to suspected or known decentration. Additionally, we included patients that underwent treatment with botulinum neurotoxin type A in general anaesthesia at the neuropediatric department of the LMU of Munich and have had recent pelvic radiographs. Patients with DDH or CP were included. Patients with other neurologic conditions, degenerative neurologic diseases, Legg-Calvé-Perthes, slipped capital epiphysis and other syndromes affecting the hip not comparable to DDH or CP were excluded. We excluded patients below 15 months of age and patients above 18 years for DDH and above 20 years for patients with CP. The threshold of 15 months was set because the aim of our method is the examination of patients with an advanced ossification process and due to the fact that we rarely perform x-rays at a lower age. The upper limits were set also due to the insufficient numbers of patients in this age group as a paediatric department. All radiographs were part of the standardized diagnostic workup either for initial assessment or as part of standardized hip surveillance in patients with known DDH or CP. The study was approved by the local ethics committee of the Ludwig Maximilians University of Munich (ethics number ethics number 20-317) and performed according to the guidelines of the Declaration of Helsinki [23]. After informed consent was obtained by the patients and their legal guardians, we examined all of the patients by standardized US as described by N. Hien [21]. Intervals longer than 3 months between x-ray and ultrasound were precluded.

### Ultrasound examination set-up

With the patient in neutral supine position and the legs in neutral rotation (knee caps at the most ventral position) and neutral ab-/adduction, lateral ultrasound images were acquired. To minimize geometrical distortion the examiner has to take meticulous care to position the transducer perpendicularly to the longitudinal axis of the body and in alignment with the horizontal plane of the underlying examination couch. For ventral analysis the transducer was positioned perpendicular to the examination couch and set along the longitudinal axis of the femoral neck. The field of view had to comprise the distal portion of the lateral aspect of the ilium, the acetabular rim and the femoral head at its largest diameter.

In order to minimize systematic errors the examiners (specialists in paediatric orthopaedics or neuropaediatrics and one trained M. D. candidate) were instructed to perform the method with the utmost precision. To ensure that they had the necessary expertise, they completed a training session with the study directors.

At our outpatient clinic we used a 7,5Mhz transducer mounted on a General Electric Venue R3 (GE Medical Systems SCS, France). At the neuropediatric department we used a Philips Affiniti 50 (Philips Ultrasound Inc., WA, USA) device.

### Image analysis

**Fig. 1:**
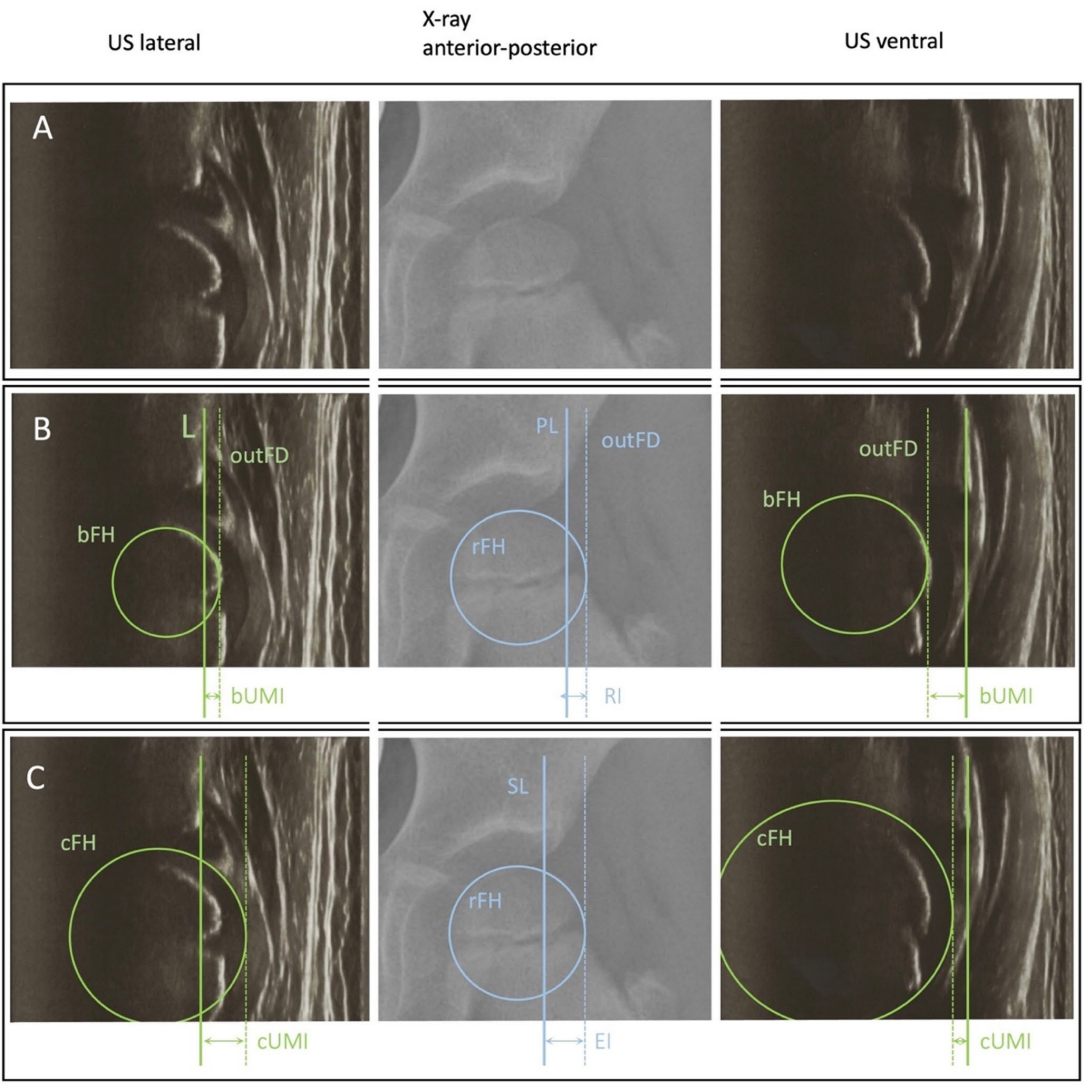
Exemplary US imaging. Left row: US lateral view; middle row: x-ray a.p. view; right row: US ventral view. (**A**) Native images. (**B**) Markers depict borders using the bony femoral head for calculating the bUMI and the radiographic RI. (**C**) Markers depict borders using the cartilaginous femoral head for calculating the cUMI; L = line through the US bony lateral edge of the acetabulum, bFH = extrapolated bony femoral head using US, cFH = extrapolated cartilaginous femoral head using US, rFH = extrapolated bony femoral head using radiography, PL = Perkins Line at the most lateral edge of the acetabulum, SL = vertical line using the lateral edge of the sourcil. dotted line = outFD (line situated at the outer femoral diameter)

Only pictures with adequate quality and all required anatomical landmarks present were admitted to the measurement protocol. For measurements on the conventional pelvic radiographs we used the Visage Client 7.1.18 (Windows, Visage Imaging). The parameters recorded were: Lateral Centre-Edge (LCE) angle, the migration percentage according to Reimers (RI) as well as the modified extrusion index (EI) [14, 24, 25]. While the RI relies on the Perkins line as lateral border of the acetabulum or the lateral aspect of the distal ilium, the EI defines the lateral border of the sourcil as the point of reference to construct the Perkins line [14] (Fig. 1).

The US images were analyzed using ImageJ 1.53k (Wayne Rasband NIH, USA). First a line (L) serving as a baseline was drawn parallel to the horizontal image plane through the bony edge of the acetabulum (A). The bony femoral epiphysis (bFH) and the cartilaginous border of the entire femoral head (cFH) were extrapolated with the circle tool. The diameter of the circles was constructed orthogonally to the baseline and the ratio of the diameter protruding vertically above the baseline was calculated and recorded. These values were then calculated as the cartilaginous ultrasonographic migration index (cUMI) and the bony ultrasonographic migration index (bUMI). This was calculated for the lateral and the ventral sections.

### Statistical analysis

For statistical analysis of this cohort study, we used SPSS (version 23, IBM, Armok, New York). *P*-values of < 0.05 were considered as statistically significant. The Chi-square test and the Mann-Whitney test were used to determine differences in means. Additionally, the Pearson correlation as well as the Kendall’s Tau correlation were used regarding the parameters of X-ray and ultrasound images. The intra- and inter-observer reliability were determined using the Intraclass Correlation Coefficient (ICC) analysis.

## Results

### Patients’ characteristics

**Fig. 2:**
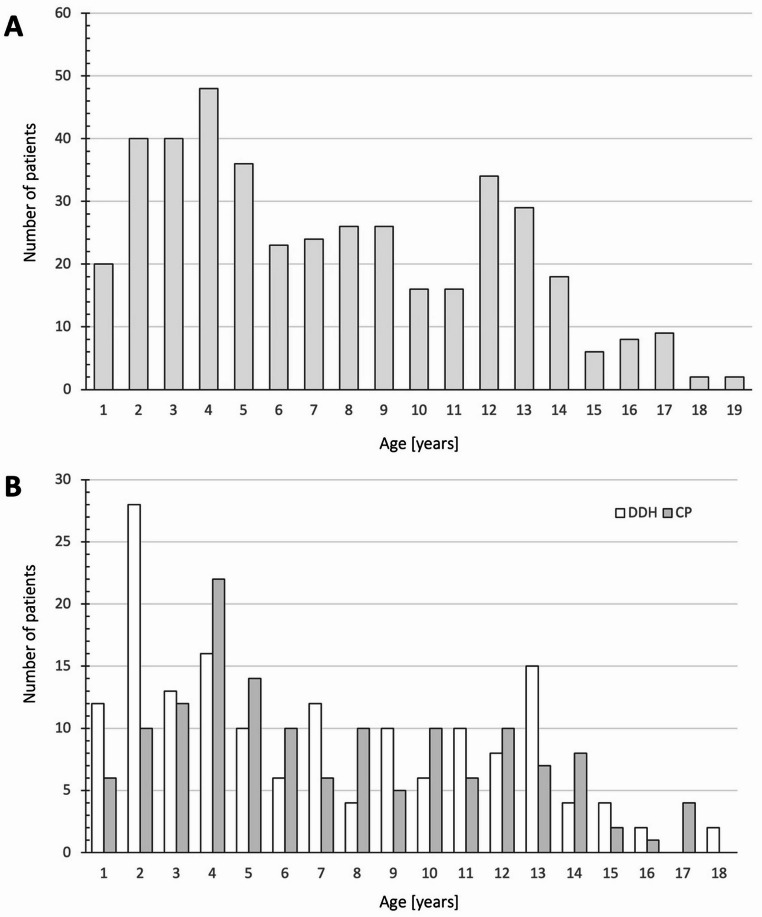
(**A**) Age distribution over-all cohort. (**B**) Age distribution depending on etiology. Graphs depict the total number of patients per age group

We enrolled 169 patients of which in 158 patients both hips could be evaluated. In six patients only the left side, in five patients only the right side could be included, rendering a total of 327 hips eligible for further analysis. 99 patients were female (58.6%), 70 patients were male. 74 patients were diagnosed with DDH (43.8%), 73 patients had CP and in 22 patients another underlying condition - not affecting the hip pathoanatomy - was present. Age distribution showed a bipolar peak pattern with increased patient count during infancy and puberty (Fig. 2).

### Lateral migration indices

**Fig. 3:**
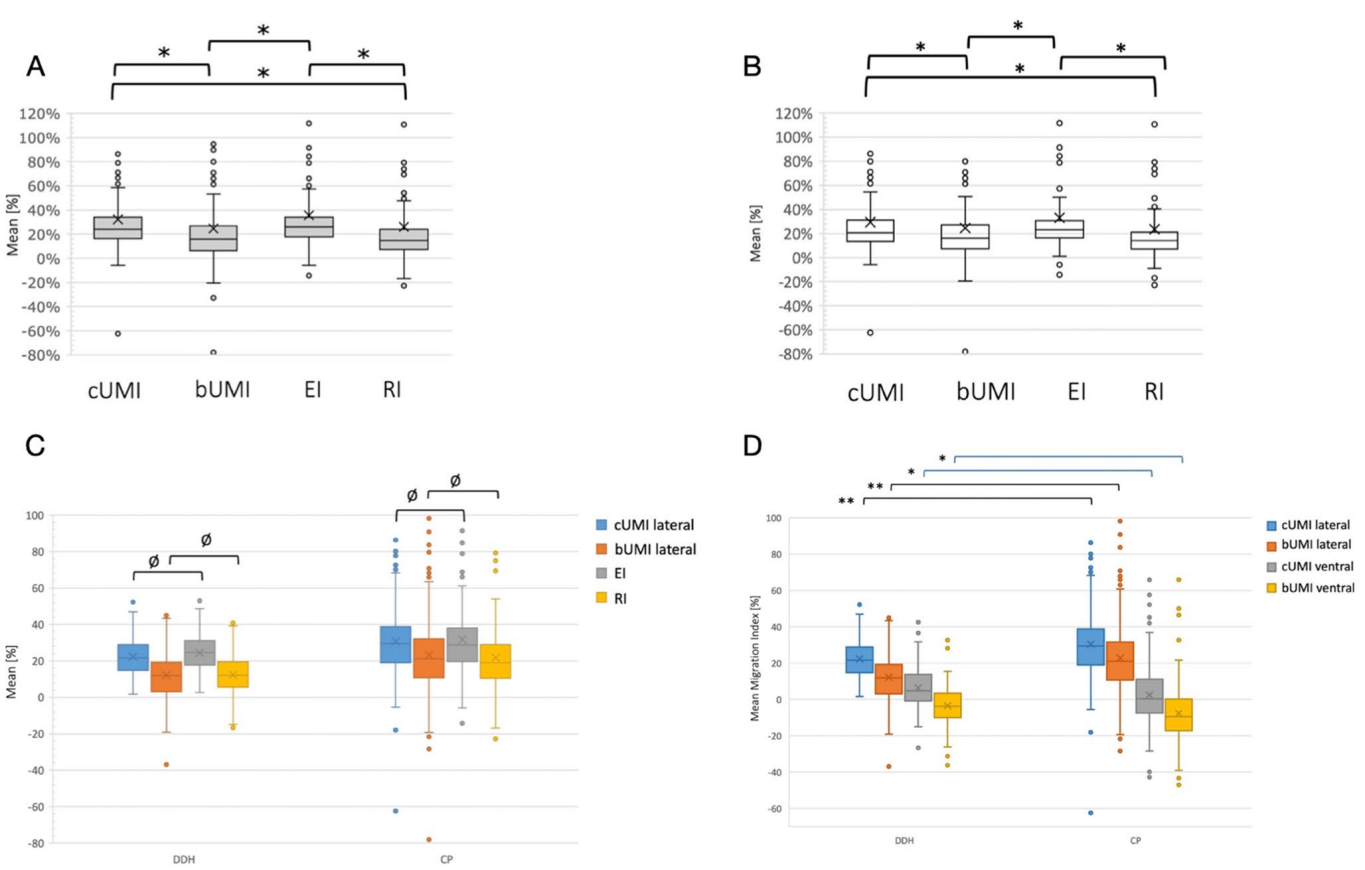
(**A**) Mean values of the overall cohort for lateral cUMI, bUMI, EI and RI. (**B**) Mean values for patients ≥ 5 years. Lower and upper limits of the box = 25th and 75th percentile, thick line in the box = mean, X = median, I-bar = maximum and minimum of the data Circle = outlier. *) *p* < 0.01 determined via unpaired t-test and Levene test; cUMI = cartilaginous ultrasonographic migration index; bUMI = bony ultrasonographic migration index; EI = Extrusion Index; RI = original Reimers Index. (**C**) Mean values of the overall cohorts for lateral cUMI, bUMI, EI and RI for CP patients and DDH patients. Lower and upper limits of the box = 25th and 75th percentile, thick line in the box = mean, X = median, I-bar = maximum and minimum of the data Circle = outlier. Ø indicates no difference between groups, all other groups showed differences with *p*-values < 0.01 determined via unpaired t-test and Levene test. (**D**) Mean values of *lateral* and *ventral* cUMI and bUMI divided by etiology (DDH vs. CP) of all patients. Lower and upper limits of the box = 25th and 75th percentile, thick line in the box = mean, X = median, I-bar = maximum and minimum of the data Circle = outlier. *) *p* < 0.05 determined via unpaired t-test and Levene test. **) *p* < 0.001

The mean lateral bUMI was considerably lower than the lateral cUMI (*p* < 0.01; Table 1; Fig. 3A and B). As expected, the radiographic original RI showed clearly lower values compared to the modified EI of (*p* < 0.01). Interestingly, no significant difference was observed between the bUMI and RI, nor was there a statistically significant difference between cUMI and EI. This finding was consistent both for the overall cohort and specifically for children over the age of five years.

**Table 1:** Results for the lateral ultrasonographic migrations indices (UMI) in relation with the according radiographic parameters

*Lateral*	Overall cohort				Patients > 5 years		
	**Mean [%]**	**Standard error of the mean** **(± SEM)**	**Number of hips**		**Mean [%]**	**Standard error of the mean** **(± SEM)**	**Number of hips**
**cUMI**	**25.9**	± 0.9	327		**23.7**	± 1.2	212
**bUMI**	**17.4**	± 1.0	327		**18.6**	± 1.3	212
**EI**	**27.7**	± 0.9	327		**25.7**	± 1.2	212
**RI**	**16.8**	± 0.9	327		**16.0**	± 1.1	212
**LCE**	**18.8[°]**	± 0.8	327		**22.5[°]**	± 1.2	212
				**Mean Age**	**10.2 [yrs]**	± 2.4	212

### Correlation of lateral ultrasound parameters and radiological migration percentages

We found a clear positive correlation in Kendall´s Tau test between the lateral cUMI and both the radiologic indices (RI and EI) as well as a clear negative correlation with Wiberg´s LCE (Table 2). Similarly, the lateral bUMI showed a clear correlation with the EI as well as even slightly higher correlation with the RI. There was only a weak correlation between the lateral bUMI and the LCE. We noted a strong agreement between RI and EI of K = 0.744 as well as the cUMI and bUMI (K = 0.711). Comparing the LCE with the radiographic indices we found a strong negative agreement for EI and a clear to strong agreement for RI. All correlations were significant *p* < 0.01 (Table 2). We found a considerable difference in LCE for all patients in contrast to lower values for patients older than five years of age (*p* < 0.01). All other measurements showed no difference when comparing these age groups (Table 1).

Upon analyzing the correlations between the described parameters for the age group over five years, a higher level of correlation was observed for all values. Supplementary Fig. 1 illustrates hip displacement in the lateral US view and x-ray a.p. .

**Table 2:**
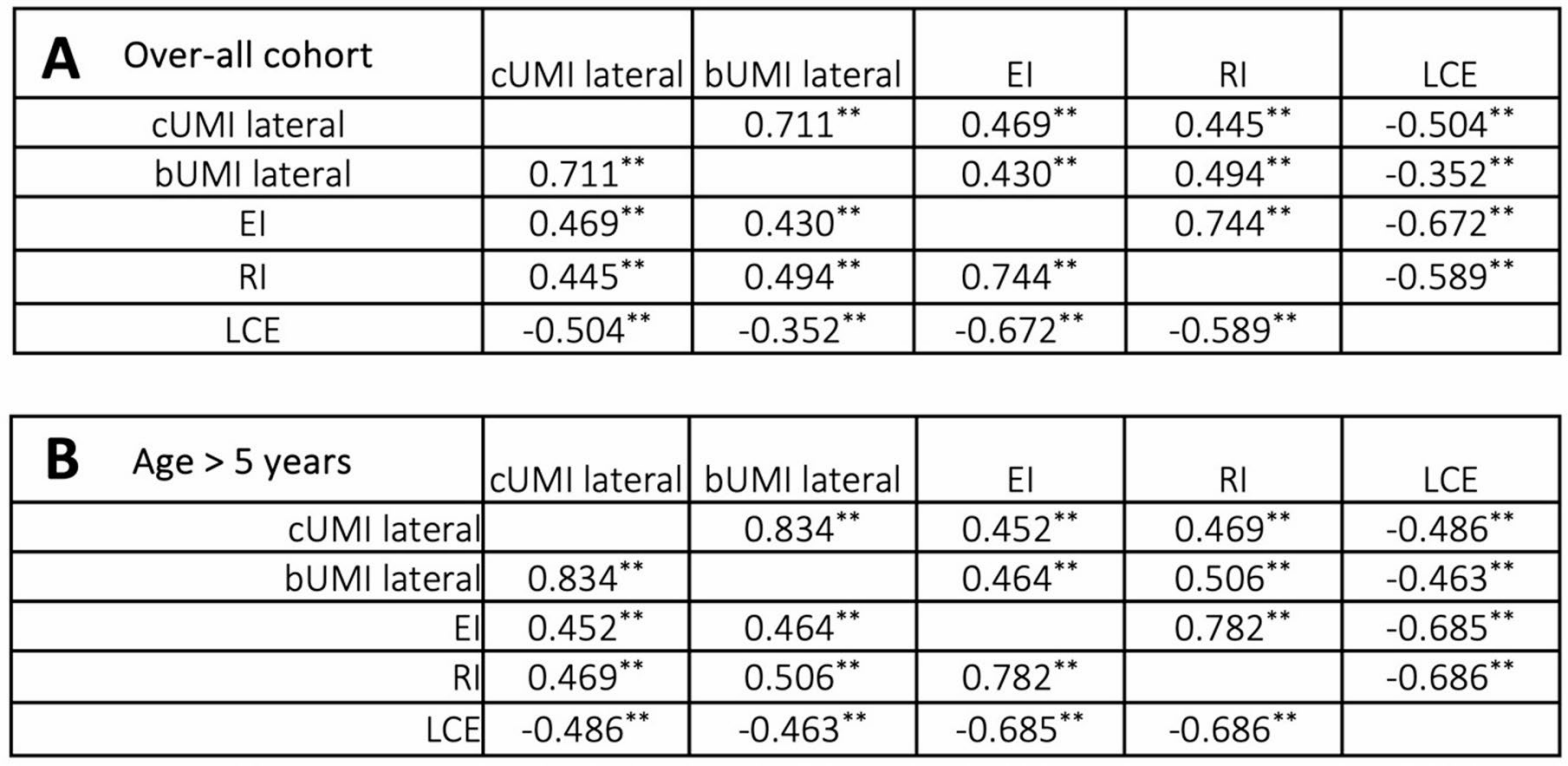
Kendall’s Tau correlation between radiologic parameters divided by age groups. A and B show the correlation coefficients. A depicts all ages, B children > 5 years of age. The following interpretation was applied: k < 0.1: no match; 0.1 < k ≤ 0.4: weak agreement; 0.4 < k ≤ 0.6: clear agreement; 0.6 < k ≦ 0.8; strong agreement; 0.81 < k ≤ 1: (almost) complete agreement. **) correlation is significant at the 0.01 level (2-tailed)

### Difference between DDH, CP for lateral and ventral UMIs

When patients were categorized based on the aetiology of their hip decentration, the same results were observed as in the overall cohort. No difference was found between the lateral cUMI and EI, as well as the lateral bUMI and RI, neither in patients with DDH nor in those with CP (Table 3; Fig. 3C). However, statistical analysis revealed significant differences between the two etiologies for each individual parameter (*p* < 0.01, Table 3; Fig. 3D). Lateral cUMI and bUMI values were significantly lower in DDH patients compared to CP patients, whereas ventral UMI values were higher in DDH patients.

**Table 3:** Mean values of each parameter divided via etiology (DDH vs. CP)

		Mean	Standard error of the mean (± SEM)	Number of hips
Age [years]	**DDH**	**7.0**	± 0.4	147
	**CP**	**7.4**	± 0.3	144
Lateral cUMI [%]	**DDH**	**22.3**	± 0.8	144
	**CP**	**30.7**	± 1.6	144
Lateral bUMI [%]	**DDH**	**12.1**	± 1.1	146
	**CP**	**23.2**	± 1.9	144
Ventral cUMI [%]	**DDH**	**6.1**	± 1.0	147
	**CP**	**2.5**	± 1.4	143
Ventral bUMI [%]	**DDH**	**-3.7**	± 0.9	147
	**CP**	**-7.7**	± 1.5	143
AC Index [°]	**DDH**	**20.4**	± 0.6	122
	**CP**	**21.1**	± 0.7	144
Ulmann-Sharp Angle [°]	**DDH**	**50.1**	± 0.4	145
	**CP**	**49.1**	± 0.5	144
LCE [°]	**DDH**	**21.0**	± 0.8	146
	**CP**	**15.5**	± 1.5	144
EI [°]	**DDH**	**24.4**	± 0.8	146
	**CP**	**31.7**	± 1.6	144
RI [°]	**DDH**	**12.4**	± 0.9	146
	**CP**	**21.8**	± 1.6	144
cUMI-bUMI	**DDH**	**12.0**	± 0.6	146
	**CP**	**9.9**	± 0.4	144

### Analysis of cartilage thickness according to age

**Fig. 4:**
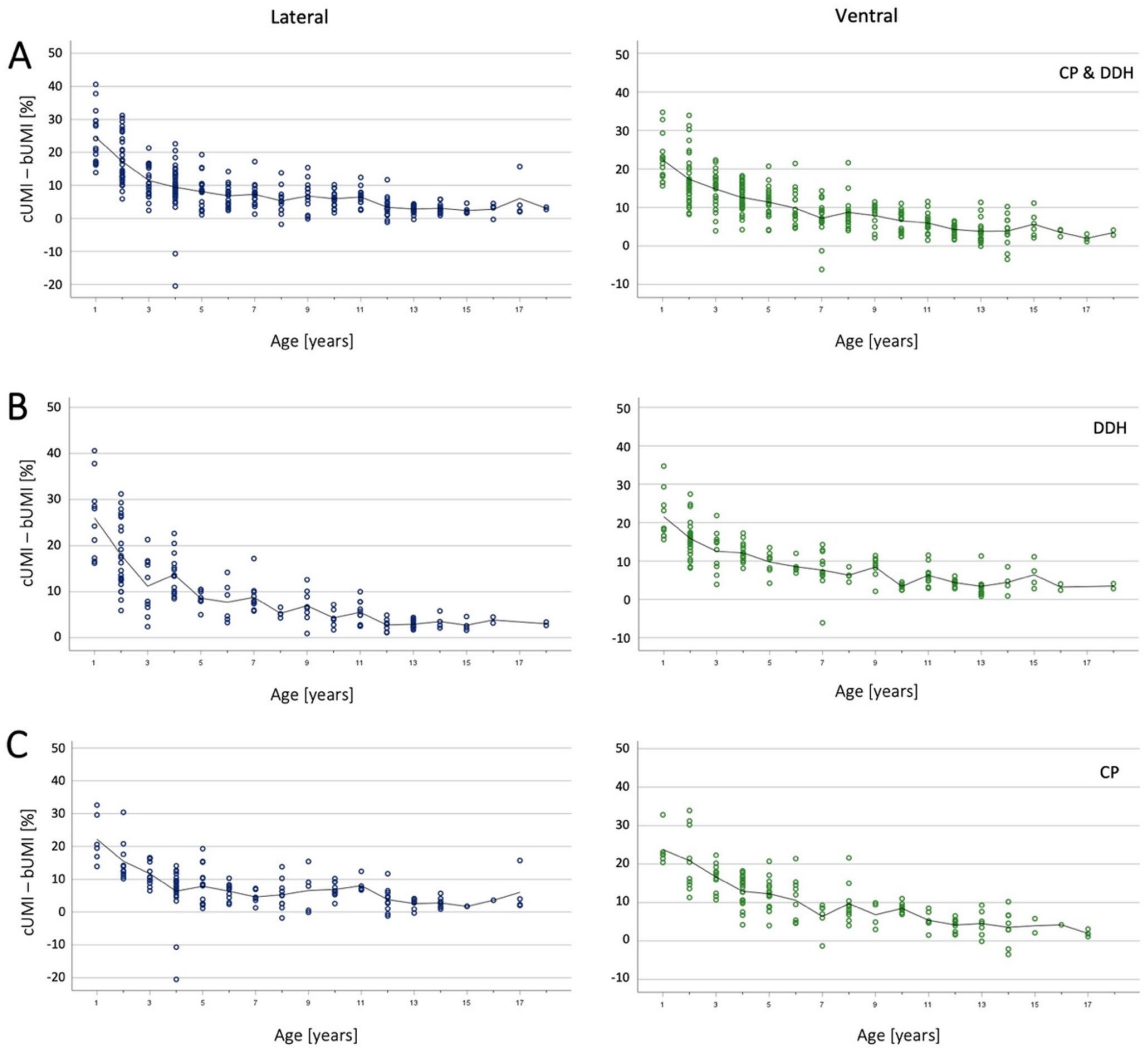
Difference between cartilaginous and bony sonographic MI measurement depending on age. Line = Mean of the difference per age group. (**A**) lateral and ventral differences for the total cohort, (**B**) for DDH patients and (**C**) for CP patients

Current literature suggests cartilage thickness at the femoral head decreases with musculoskeletal development [26]. To determine if this could also be confirmed sonographically, we conducted an age-dependent analysis of cUMI and bUMI (cUMI-bUMI [%]), as we assumed that the smaller the cartilage thickness, the smaller the difference cUMI-bUMI due to geometric-mathematical conditions. We calculated the difference between the two values and then averaged this difference for each age group (Fig. 4). The results show a trend line that tends towards zero with increasing age, corresponding to decreasing cartilage thickness. Furthermore, a relatively wide variation in the first five years of life can be observed. Starting at the age of five years, the difference cUMI-bUMI stabilized. This finding applied to both lateral and ventral parameters, as well as to each aetiology.

### Inter- and intrarater reliability

We determined the inter- and intrarater reliability for the new method, focusing on the lateral and ventral ultrasound parameters. Both analyses revealed a highly significant inter- and intraclasscoefficient (ICC; Table 4).

**Table 4:** Inter- and intraclass coefficients for *Lateral* and *Ventral* cUMI and bUMI. Upper and lower bounds of the confidence intervals (CI) as well as *p*-values are depicted

	ICC	95% Confidence Interval		Sig. *p* =
		Lower bound	Upper bound	
Int***er***rater-Reliability				
***Lateral*** **cUMI**	0.923	0.907	0.936	< 0.001
***Lateral*** **bUMI**	0.932	0.917	0.945	< 0.001
***Ventral*** **cUMI**	0.895	0.870	0.915	< 0.001
***Ventral*** **bUMI**	0.877	0.850	0.900	< 0.001
Int***ra***rater-Reliability				
***Lateral*** **cUMI**	0.895	0.980	0.987	< 0.001
***Lateral*** **bUMI**	0.877	0.984	0.989	< 0.001
***Ventral*** **cUMI**	0.861	0.827	0.889	< 0.001
***Ventral*** **bUMI**	0.891	0.863	0.913	< 0.001

## Discussion

The aim of our study was to evaluate the efficacy and reliability of US for detecting femoral head decentration and to compare US with conventional radiographic methods. The overall goal was to find a less harmful yet accurate diagnostic screening tool that minimizes radiation exposure in these patients. We found pathoanatomical differences when analyzing the different age groups and the different etiologies. Our results suggest that US may be a viable alternative to assess hip decentration in children with CP and DDH.

### Correlation of US with standard radiographic parameters for hip decentration

Our study showed that the lateral bUMI was significantly lower than cUMI, which is not surprising regarding the underlying anatomy. Terjesen et al. have described the lateral head distance (LHD) and the ventral head distance (VHD) using the bony margin of the femoral head. The authors have used absolute values in millimeters and suggested age-dependent normal values. In our view, this is not suitable for the growing musculoskeletal system, as they lack practicability in the daily routine. Relative values are often comparable over time and during growth, as we have seen in the case of anterior knee pain [27]. Our observation that the original RI values were lower than the modified EI values is consistent with previous research findings (Parrott, Boyd et al., 2002; Wyatt and Beck, 2018) and confirms the reliability of our radiological measurements. Kay et al. have used a parameter comparable to the bUMI but used 3-dimensional US [19]. Until now, these methods have been much more complex and difficult to implement in daily routine. The authors used RI as radiographic reference. Our results also showed a good correlation between RI and bUMI (K = 0.494). This is weaker compared to Kay`s method, but still a clear agreement. However, it should be taken into account that their method was far from being able to include all patients in the measurement protocol, as the method’s demands on the images were very high. Nevertheless, our method is also prone to error due to the extrapolation of the circular femoral head in only one plane.

The lack of significant differences between bUMI and RI and between cUMI and EI indicates that US can reliably reproduce radiographic results. The correlation coefficients (K = 0.744 for RI and EI and K = 0.711 for cUMI and bUMI) show that these measurements are highly concordant. bUMI and RI use the same anatomical landmarks, namely the outermost lateral edge of the acetabulum and the bony margin of the femoral head [14]. The finding that there is no significant difference between the cUMI and the EI may be coincidental - although both methods use different landmarks at a greater distance compared to bUMI and RI - but it is a sign of systematic accuracy of the method. Kulkarni et al. have shown that the three most commonly used radiographic parameters for hip decentration already correlate well with each other [26, 28].

Our study also showed a clear negative correlation between the lateral UMI and the LCE angle. This indicates that the US parameters accurately reflect the radiographic findings [13, 22].

Furthermore, our results show that the correlation between two parameters is strong when both parameters are measured with the same imaging tool. When comparing parameters from US and X-ray, it is important to remember that the landmarks are displayed with different degrees of clarity and that the planes may not exactly overlap.

### Age-dependent analysis and cartilage thickness

Castriota-Scanderbeg et al. showed, that cartilage thickness can be determined effectively via US [29]. Nevertheless, they did not investigate age dependent differences. Although the children aged five years and under had a thicker cartilaginous surface of the femoral head in our study, this concordance is crucial as younger children, who are likely to undergo imaging more frequently (Fig. 2), benefit most from lower radiation exposure. Our results show that US remains accurate and reliable as children grow older and their hip anatomy changes. Our age-dependent analysis revealed significant fluctuations in cartilage thickness especially during the first five years of life, with stabilization occurring at around five years of age. This pattern reflects the dynamic changes in musculoskeletal development. Wittoek et al. already showed, that cartilage thickness decreases during growth when performing an US analysis of 500 healthy children [26]. Schmaranzer et al. analyzed the thickness of the cartilaginous limbus of the acetabulum in DDH using MRI and found that it has a prognostic value for residual DDH after open or closed reduction in infants [30, 31]. In our study, we could not analyze the thickness of the acetabular cartilage due to the nature of the technique. Nevertheless, we found no differences in cartilage thickness when we divided the patients according to etiology.

The stabilization of cartilage thickness after five years of age may suggest a potential age-related benchmark for the reliability of our method that may be useful for clinicians to monitor hip development in pediatric patients. Therefore, we recommend using this tool for hip surveillance starting at a patient age of five years, although we also see the usefulness of this method for younger children, especially in regards to radiation exposure. Further analysis with a larger study population at this age group will determine if this benchmark is set at the right age.

### Differences between the etiologies DDH and CP

Significant differences in lateral and ventral cUMI, bUMI were found in our study when comparing patients with either DDH or CP. Although our study does not reflect a fully age-matched cohort – as well as a non-coherent distribution of GMCF levels (Gross Motor Function Classification System) - these differences may reflect variations in hip joint morphology and stability between the two conditions.

When considering the etiology- and age-specific differences, the patient structure of the study must be taken into account. The patient population reflects the patients who received scheduled radiologic follow-up in our outpatient clinics according to the severity of their disease. At least in the study population, patients with CP are affected by more severe hip decentration than the general population or patients with DDH. Since the follow-up examinations are only scheduled in cases of corresponding disease severity and possible treatment consequences, our results do not allow conclusions to be drawn about the extent of hip decentration in the (etiology- and age-specific) patient populations as a whole.

However, the finding that lateral cUMI and bUMI values were significantly lower in DDH patients compared to CP patients, while ventral UMI values were higher in DDH patients, emphasizes the need to consider anatomical and biomechanical differences when interpreting not only our US results when analyzing CP or DDH. Our results are somewhat surprising as DDH hips are normally decentered dorsally and CP hips ventrally. Nevertheless, the ability to consistently measure cartilage thickness and detect hip decentration in different anatomical planes and conditions increases the utility of US. However, neither of the standardized types of sectional imaging (MRI or CT) are part of standardized screening. Therefore, we could not compare our results with the expected results of these imaging techniques. Future studies are desirable to enable a more precise using an applicable three-dimensional analysis.

### Reliability

The high inter- and intrarater reliability of the new US method, resulting from the intraclass correlation coefficients (ICC) for the lateral and ventral parameters, underlines the robustness of US as a diagnostic tool. Pham et al. have also shown high repeatability for hip US in a low patient number but did not compare different aetiologies and different radiographic measurements and anatomical landmarks such as the bony and cartilaginous diameters of the femoral head [32]. Nevertheless, US has its own limitations. Image quality depends on various factors, such as the body surface and nutritional status. Image quality is a prerequisite and may not be sufficient for analysis in some cases. Additionally, variations in interrater agreement are well recognized in ultrasound diagnostics across different medical specialties compared to other imaging modalities. These discrepancies often depend on the level of experience of the examiner [33–36].

Therefore, further verification of our results in multi-center studies is desirable in order to investigate the efficacy of our method. This is essential for therapeutic and diagnostic recommendations as well as for health economic evaluation as US may result in higher costs due to time consuming workflow if not trained and established effectively.

## Conclusion

The high correlation between US and radiographic parameters and the significant inter- and intrarater reliability support the use of US as a safe, non-invasive diagnostic tool that reduces radiation exposure. Multi-centre and above all follow-up studies are necessary to establish US examinations for monitoring the progress of hip decentration.

The integration of advanced imaging technologies, such as three-dimensional US and automated image analysis software, could improve the accuracy and efficiency of US examinations [37]. These innovations have the potential to streamline diagnostic workflows and enable more detailed and comprehensive assessments of hip joint morphology and development.

## Supplementary Information

Below is the link to the electronic supplementary material.

Supplementary Material 1

## Data Availability

Data is provided within the manuscript or supplementary information files. The raw data will be provided by the corresponding author upon reasonable request.
