# Supplementary material for: Ultrasound is a suitable radiation-free alternative for hip surveillance in children with cerebral palsy or developmental dysplasia of the hip older than one year
Source: Int Orthop. 2025 Dec 4;50(1):189–200. doi: 10.1007/s00264-025-06701-2 (PMC12881170; doi:10.1007/s00264-025-06701-2)
Supplement: Supplementary file 1 — Supplementary Material 1 [file 264_2025_6701_MOESM1_ESM.docx]

**Supplementaries**

**Supplementary Table 1.** Correlation between radiologic parameters divided by groups (DDH vs CP). A and C depict Pearson correlation. The following interpretation was applied: k<0.1: no match; 0.1< k ≤ 0.4: weak agreement; 0.4< k ≤ 0.6: clear agreement; 0.6<k≦0.8; strong agreement; 0.81< k ≤ 1: (almost) complete agreement. **. Correlation is significant at the 0.01 level (2-tailed). B and D depict Kendall’s Tau.


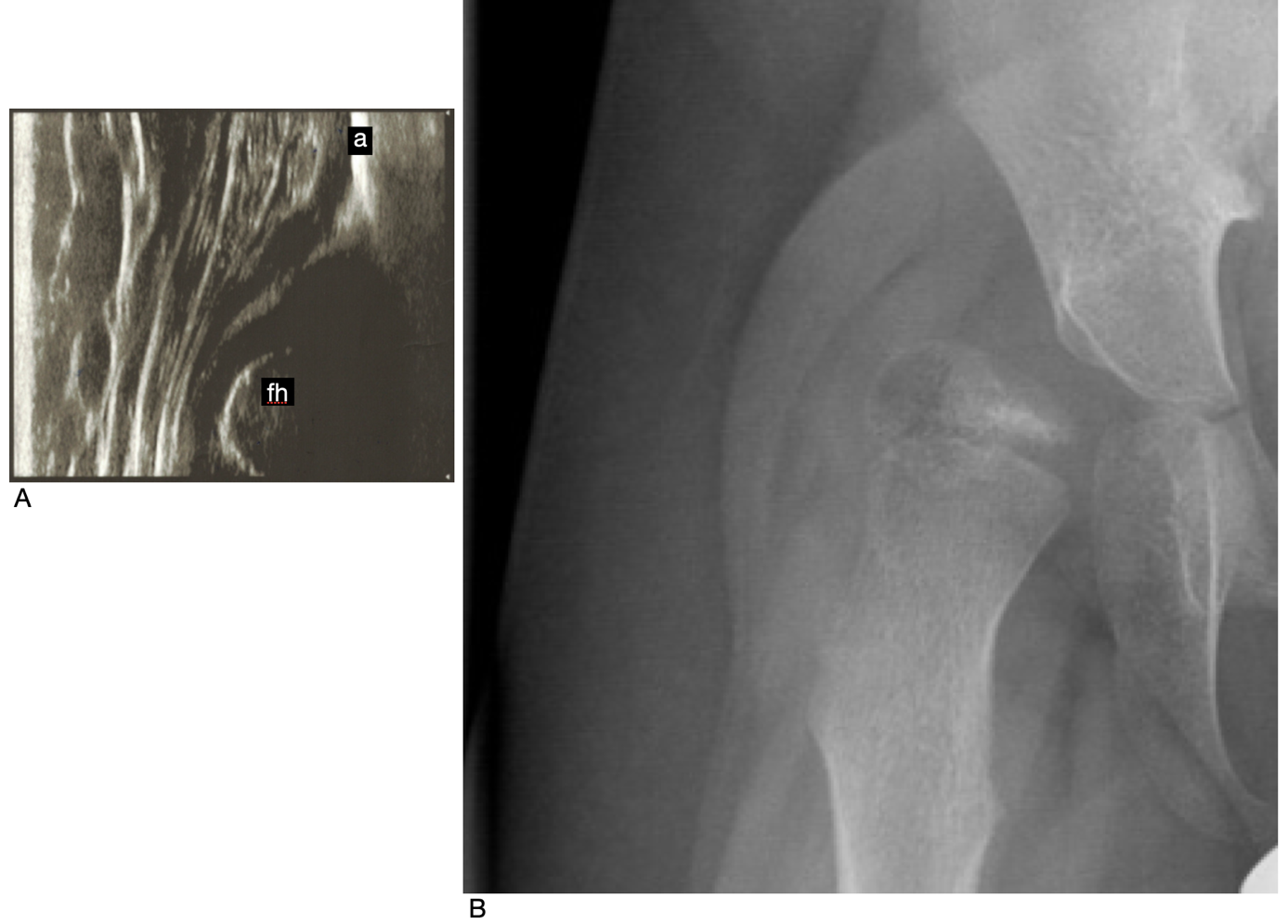


**Supplementary Figure 1.** Right hip of a 4-year-old male patient with CP and hip displacement. (A) US lateral view, (a) acetabulum, (fh) femoral head, (B) corresponding x-ray a. p. .
